# MAIA—A machine learning assisted image annotation method for environmental monitoring and exploration

**DOI:** 10.1371/journal.pone.0207498

**Published:** 2018-11-16

**Authors:** Martin Zurowietz, Daniel Langenkämper, Brett Hosking, Henry A. Ruhl, Tim W. Nattkemper

**Affiliations:** 1 Biodata Mining Group, Faculty of Technology, Bielefeld University, Bielefeld, Germany; 2 National Oceanography Centre, University of Southampton Waterfront Campus, Southampton, United Kingdom; 3 Monterey Bay Aquarium Research Institute, Moss Landing, California, United States of America; State University of New York at Buffalo, UNITED STATES

## Abstract

Digital imaging has become one of the most important techniques in environmental monitoring and exploration. In the case of the marine environment, mobile platforms such as autonomous underwater vehicles (AUVs) are now equipped with high-resolution cameras to capture huge collections of images from the seabed. However, the timely evaluation of all these images presents a bottleneck problem as tens of thousands or more images can be collected during a single dive. This makes computational support for marine image analysis essential. Computer-aided analysis of environmental images (and marine images in particular) with machine learning algorithms is promising, but challenging and different to other imaging domains because training data and class labels cannot be collected as efficiently and comprehensively as in other areas. In this paper, we present Machine learning Assisted Image Annotation (MAIA), a new image annotation method for environmental monitoring and exploration that overcomes the obstacle of missing training data. The method uses a combination of autoencoder networks and Mask Region-based Convolutional Neural Network (Mask R-CNN), which allows human observers to annotate large image collections much faster than before. We evaluated the method with three marine image datasets featuring different types of background, imaging equipment and object classes. Using MAIA, we were able to annotate objects of interest with an average recall of 84.1% more than twice as fast as compared to “traditional” annotation methods, which are purely based on software-supported direct visual inspection and manual annotation. The speed gain increases proportionally with the size of a dataset. The MAIA approach represents a substantial improvement on the path to greater efficiency in the annotation of large benthic image collections.

## 1 Introduction

With the establishment of deep learning methods, the task of image classification seems to be solved for many computer vision scenarios, sometimes even outperforming human experts on benchmark datasets [1]. However, automatic object detection is still below expert performance in most contexts and has gained more and more attention in recent years. Although applications for automatic object detection exist, they focus mostly on images that show everyday objects from scenes on land, neglecting the image domain of environmental monitoring data such as marine imagery.

Imaging in marine science is a noninvasive method to monitor and explore marine habitats (e.g. estimating biodiversity or ecological management). Due to advances in marine technology (marine digital imaging, autonomous underwater vehicles (AUVs), remotely operated vehicles (ROVs), etc.) vast amounts of image data can be acquired. The evaluation of these datasets is usually done by manual image annotation with dedicated tools like SQUIDLE+ (http://squidle.org) or the Bio-Image Indexing and Graphical Labelling Environment (BIIGLE) 2.0 [2]. While in computer science research areas such as media informatics or image databases, image annotation refers to the assignment of semantics to whole images, describing the content on a high level, image annotation in this context refers to the assignment of meaning (i.e. a class label selected from a given taxonomy) to a region of an image [3–5]. Even with dedicated tools, the purely manual approach to this kind of image annotation is still a time-consuming and error-prone task [3, 4]. In addition, owing to the complexity and diversity of organisms found in marine imagery, only domain experts are usually able to provide object detection and class labels with sufficient quality (i.e. sufficient inter-/intraobserver agreement and accuracy). This contrasts the situation of established computer vision benchmark image datasets like MS COCO [6], where crowdsourcing can be employed to generate large amounts of image annotations for everyday objects.

During manual image annotation, most of the time is spent on locating objects of interest (OOI) rather than assigning a correct class label for the object [3]. Moreover, OOI that were missed during image annotation are a key part of the differences in annotation quality between human observers [5]. Over the years, some methods have been proposed to assist in underwater object detection, like ranking images or image regions by saliency [7] or detecting interesting events in video streams [8]. An object detection method that efficiently and effectively generates annotation candidates (i.e. regions showing potential OOI) for marine images to increase the speed and volume of manual annotations, has yet to be developed.

In this paper, we present the Machine learning Assisted Image Annotation method (MAIA). The method aims to speed up manual image annotation by automating the process of object detection and instance segmentation of OOI in the images. Liberated from the task of object detection, human observers can concentrate purely on the classification of OOI. In the context of marine biology and environmental sciences, OOI could be particular species of interest or general megafauna like starfishes, holothurians or sponges. MAIA is based on a combination of two machine learning methods: Autoencoder networks (AEN) [9] and fully convolutional networks (FCN) [10].

Fully convolutional networks were introduced by Long et al. [10], who adapted a neural network for image classification into a fully convolutional network that performs semantic segmentation of an image. One of the more recent variants of FCNs is the Mask Region-based Convolutional Neural Network (Mask R-CNN) [11], which is capable of instance segmentation. Instance segmentation generates a bounding box, a class label and a pixel-accurate segmentation mask for each OOI in an image. Mask R-CNN can achieve impressive results, condition to the availability of a sufficient number of annotated training data samples, i.e. images with marked objects. Since the collection of training data in marine sciences is considerably expensive as it requires substantial experience and academic education, we developed MAIA to reduce time and effort on this end. In MAIA, Mask R-CNN is employed for instance segmentation and the resulting segmentation masks can be used as annotation candidates. To the best of our knowledge, we are the first to employ Mask R-CNN in the context of marine environmental monitoring and exploration.

As the supervised Mask R-CNN needs a sufficient amount of training data, we apply unsupervised AEN for novelty detection to efficiently and effectively generate training proposals, which are points of potential OOI in the images that could be considered for training a Mask R-CNN model. Based on the assumption that OOI are rare in the images, “background” pixels of the seabed can be regarded as common patterns and “interesting” pixels of objects are regarded as novel patterns. The concept of AEN was first presented by Baldi and Hornik in 1989 [9] and has been used in various contexts like dimensionality reduction [12, 13], human pose recovery [14] or cell nuclei detection [15]. AEN have been previously used for novelty detection as well [16, 17] but not in the context of FCN training data collection or environmental imaging.

The contributions of this paper can be summarized as follows: (1) We present a machine learning assisted method for image annotation that allows faster manual image annotation than methods that were used before, (2) we are the first to present the use of Mask R-CNN in the context of marine environmental monitoring and exploration, and (3) we present a detailed analysis of the manual annotation speed with three image collections featuring different types of background, imaging equipment and object classes.

In the following, MAIA is first described at the methodological level, dividing the method into four different stages to be performed one after the other (see Section 2). To assess the performance of the method regarding efficiency (i.e. time required per annotation) and accuracy (i.e. evaluation of statistics on correct and incorrect annotations), we have applied MAIA to three different datasets, referred to as JC77, SO242 and PAP, that are described in Section 3. The results of these applications are summarized in Section 4 and discussed in Section 5 regarding the dataset-specific performances and general observations. The dataset of the results can be accessed at [18]. Visual exploration of the results is possible in BIIGLE 2.0 at https://biigle.de/projects/139 using the login maia@example.com and the password MAIApaper. The manuscript ends with a short conclusion about the relevance of our results and the MAIA method for benthic image annotation.

## 2 Methods

**Fig 1:**
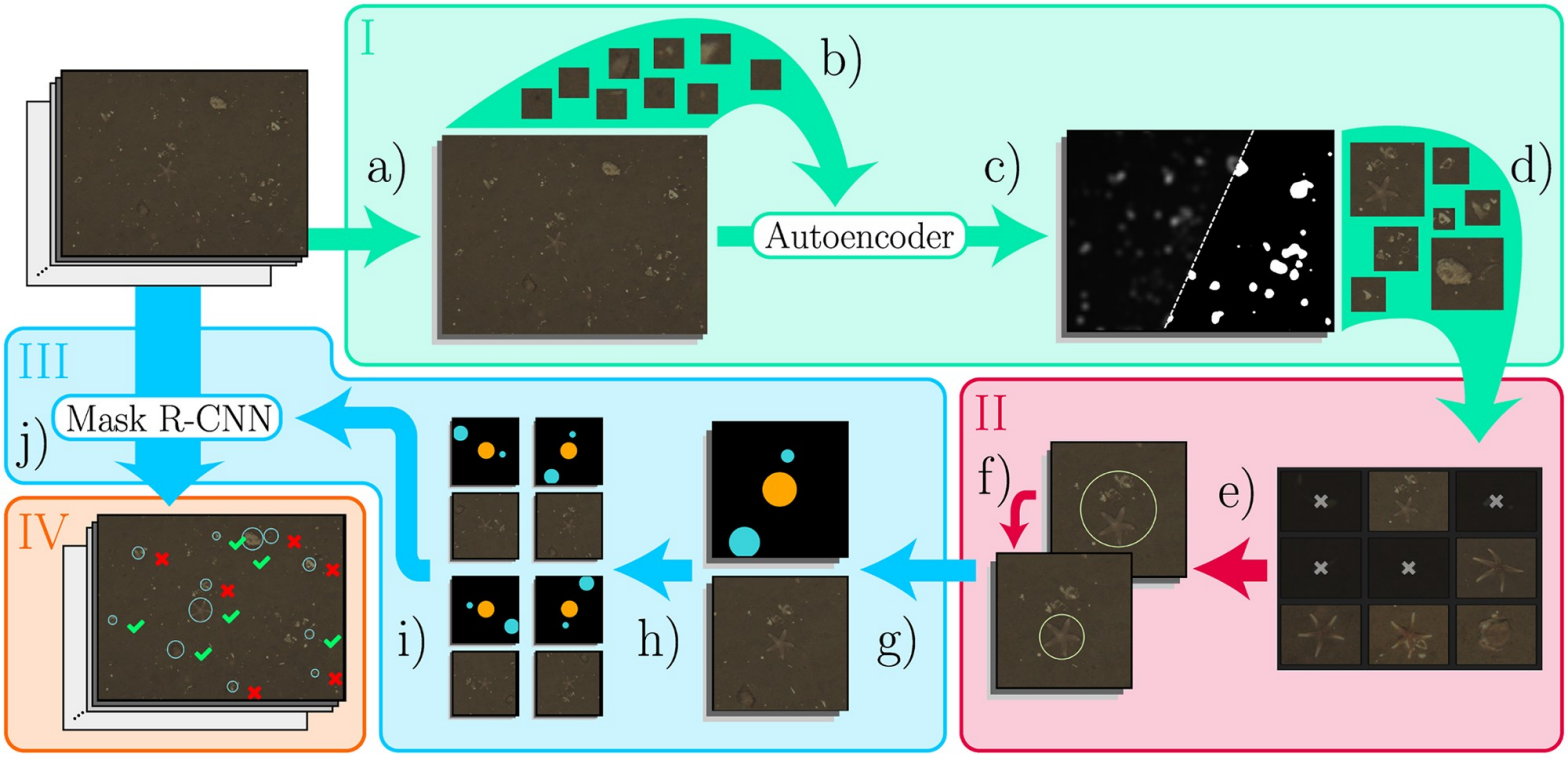
An overview of MAIA. Steps of the four stages are shown in different colors. a) Extraction of a random subset of the images. b) Training of the autoencoder network (AEN) with random image patches extracted from the subset. c) Application of AEN. d) Thresholding of the novelty maps and extraction of interesting regions as training proposals *T*_*p*_. e) Manual filtering of *T*_*p*_. f) Manual refinement of filtered *T*_*p*_. g) Creation of the training dataset *T*_*s*_. h) Boosting of the training dataset. i) Training of Mask R-CNN. j) Detection with Mask R-CNN on the whole image dataset and review of annotation candidates *A*_*c*_.

MAIA consists of four consecutive stages (see Fig 1), all of which are described in the following sections. In Stage I, unsupervised novelty detection generates an initial set of training proposals *T*_*p*_, which are image patches (i.e. regions of an image) showing patterns different from the background. These training proposals contain potential OOI that could be used in a training dataset for the instance segmentation. In Stage II, *T*_*p*_ is manually filtered to keep only those training proposals that actually show OOI relevant to the application context. In addition to that, the training proposals are manually refined with regard to their centroids and size, resulting in the dataset of training samples *T*_*s*_ ⊆ *T*_*p*_. In Stage III, the set *T*_*s*_ is used to train a Mask R-CNN model for instance segmentation which is subsequently applied to produce a set *A*_*c*_ of annotation candidates for a whole image dataset. In Stage IV, these candidates are manually reviewed to remove false positives for the final set *A* ⊆ *A*_*c*_ of detected objects and their bounding boxes.

### Stage I: Novelty detection with AEN

**Fig 2:**
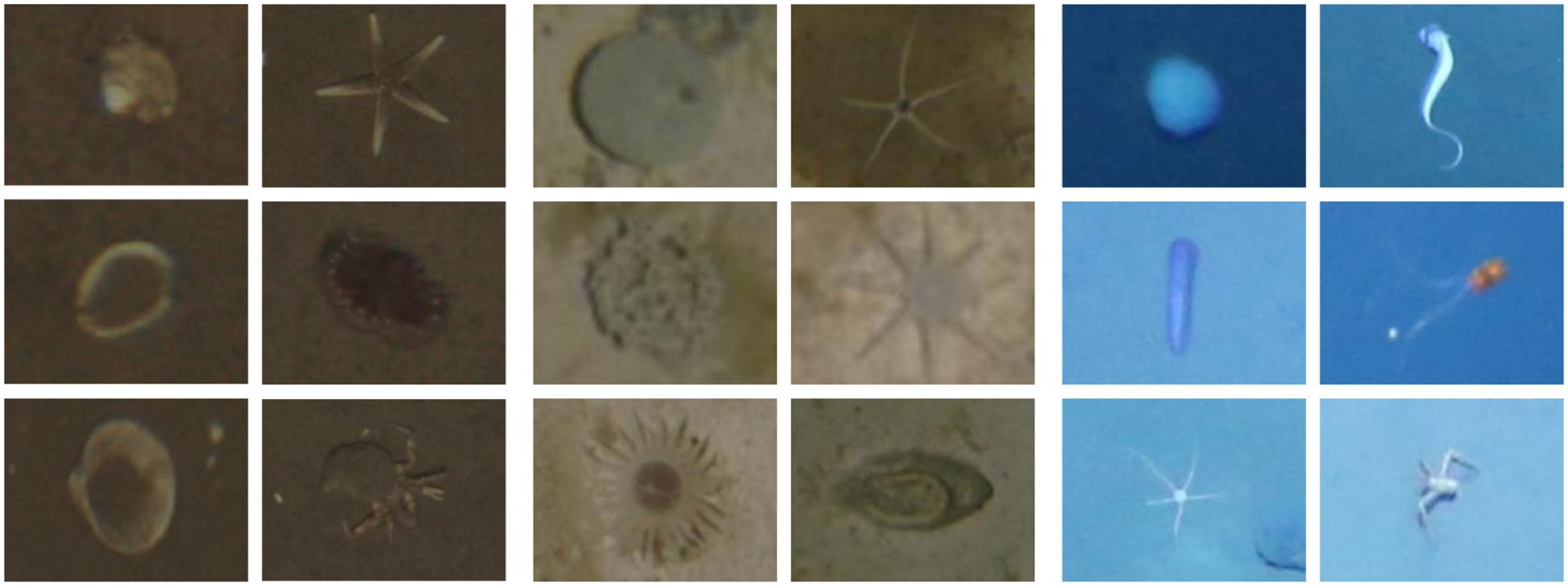
Examples of frequent OOI in the JC77 (columns 1-2), PAP (columns 3-4) and SO242 (columns 5-6) datasets.

A dataset of images {*I*_*i*_} from an AUV or ROV dive can feature different kinds of background patterns due to changes in sediment type, illumination or geological characteristics of the seabed (e.g. see SO242 in Fig 2). Thus, the images {*I*_*i*_} from one dataset are first grouped into different clusters {*U*_*k*_}_*k* = 1, …, *K*_, each one representing images with a similar background. To this end, each *I*_*i*_ is mapped to a feature vector *v*_*i*_ which is used for *k*-means clustering of all images. The features were determined empirically as a combination of principal component projection coordinates and an entropy measure. Details are given in the supporting information (see S1 Text). Each resulting cluster *U*_*k*_ contains images featuring global similarities, mostly dependent on the background sediment. For each cluster, one AEN is trained, all sharing the same architecture.

Each of the *K* AEN consists of three fully connected layers. From the first to the second layer (the latent layer), an input image patch $x\in R^{r}$ is encoded to a lower dimensional latent representation $l\in R^{s}$ with *s* < *r*, where *W* is a matrix of weights, *b* is a vector of biases and *σ*(⋅) is the softplus transfer function (see Eqs 1 and 3). If the RGB input image patch has a size of *r*_*e*_ × *r*_*e*_ pixels, the input layer dimension is $r=3\cdot r_{e}^{2}$. From the second to the third layer, the latent representation is decoded to the output patch $x^{\prime }\in R^{r}$, where *W*′ is a matrix of weights and *b*′ is a vector of biases (see Eq 2).

$$\begin{array}{ccc} l & = & \sigma (Wx+b) \end{array}$$ (1)

$$\begin{array}{ccc} x^{\prime } & = & \sigma (W^{\prime }l+b^{\prime }) \end{array}$$ (2)

$$\begin{array}{ccc} \sigma (x) & = & \log(1+\text{exp}\;x) \end{array}$$ (3)

The AEN is then trained to generate *x*′ as a reconstruction of *x* by minimizing the reconstruction error F through backpropagation (see Eq 4).

$$\begin{array}{c} F\left(x,x^{\prime }\right)={\left(x-x^{\prime }\right)}^{2} \end{array}$$ (4)

As motivated above, we consider image patches that show the pure seabed or background as common patterns and patches that show an interesting object (like a shell or a starfish) as novelties. This definition is based on the assumption that interesting objects are rare in deep-sea image datasets. Those rare objects would appear as novelties and patches featuring such a pattern also feature a large reconstruction error F. By applying a threshold to the reconstruction error, background pixels can be separated from pixels that belong to an OOI.

To train the AEN for one cluster of images *U*_*k*_, we sample 10^4^ image patches, each of the form $x=(p_{1}^{\left(r\right)},p_{1}^{\left(g\right)},p_{1}^{\left(b\right)},\ldots ,p_{r/3}^{\left(b\right)})$ and of size *r* = 39 × 39 × 3 (i.e. a 39 pixel square of RGB values) randomly from *U*_*k*_ (see Fig 1b). The RGB values of each pixel *p*_*j*_ are “flattened” to the *r*-dimensional input *x*. The patch size was determined with a parameter search for good detection performance (see Section 4). We choose the dimension of the latent representation of the AEN as *s* = [0.1*r*], with [⋅] as rounding operation. The “compression factor” of 0.1, again, was determined with a parameter search (see Section 4). To accelerate convergence during training, we use Xavier initialization [19] of the weights *W* of the encoding layer and Adam optimization [20] with a fixed learning rate of 10^−3^. We train for 100 epochs with a minibatch size of 128. Training takes less than a minute per image cluster for all datasets, using TensorFlow [21] on a single NVIDIA Titan X. We refer to the trained AEN of an image cluster *U*_*k*_ with the term data-driven background model DBM_*k*_.

**Fig 3:**
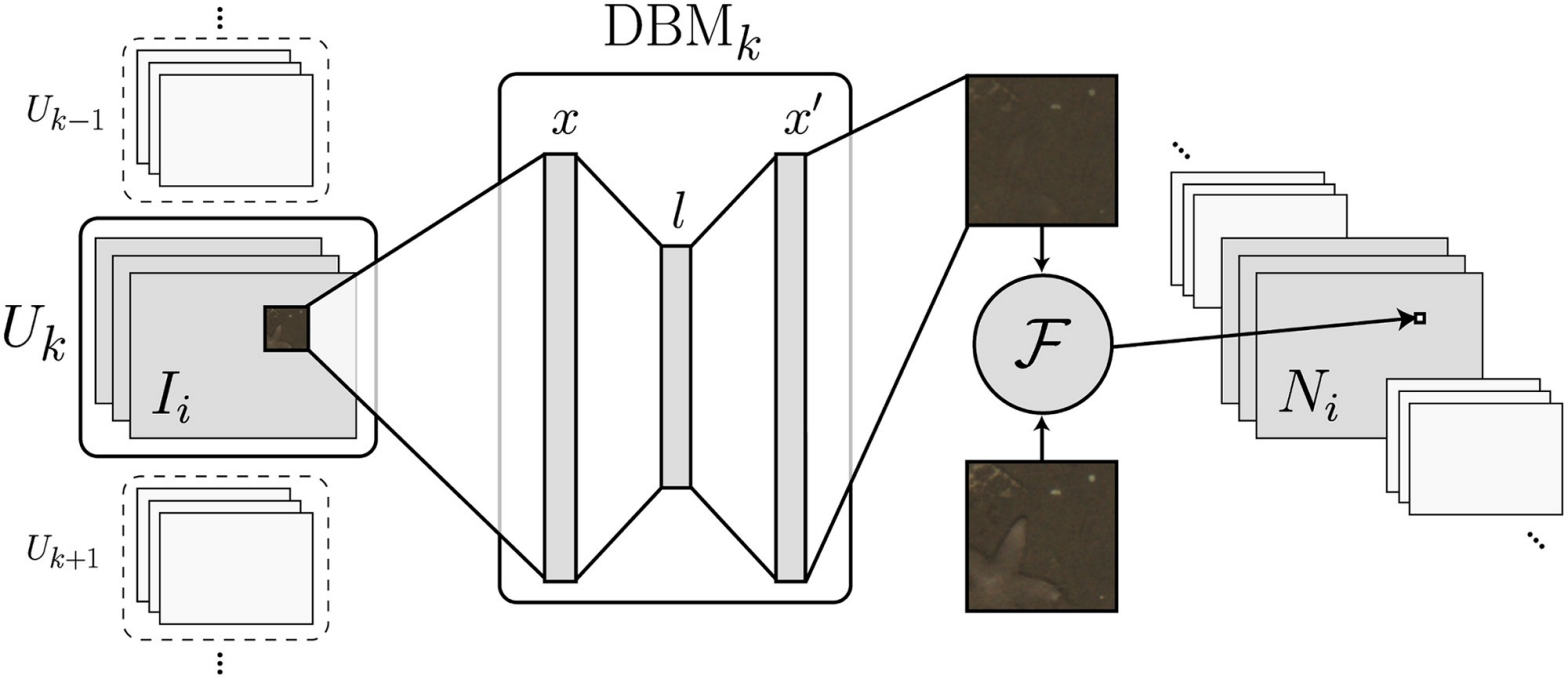
The data-driven background model (DBM_*k*_) is applied like a convolution to the input image *I*_*i*_ ∈ *U*_*k*_. The image patch is flattened to the input *x*. The input *x* is compressed to the latent representation *l* and then reconstructed to the output *x*′. The reconstruction error $F(x,x^{\prime })$ determines the value in the novelty map *N*_*i*_ at the position of each patch.

Each of the *K* DBM_*k*_ is applied to the images *I*_*i*_ ∈ *U*_*k*_, processing each possible image patch of *r*_*e*_ × *r*_*e*_ pixels (see Fig 3). This can be compared to a convolution operation. We choose a stride of two for the convolution, since it had no impact on detection performance but a higher computational efficiency when compared to a stride of one. The higher stride results in an output of lower resolution than the original image, so the output is upscaled back to the original resolution of the image *I*_*i*_ using bilinear interpolation. Owing to GPU memory constraints, a DBM_*k*_ is consecutively applied to horizontal image slices that are stitched together afterward. The reconstruction error $F(x,x^{\prime })$ of each image patch *x* is stored as the “novelty score” at the pixel coordinate of the center of the patch, resulting in the “novelty map” *N*_*i*_ for each image *I*_*i*_. The novelty map is convolved with an all-ones kernel of the same size as the training patches, which acts as a dilation to smooth the boundaries of regions with a high novelty score (see Fig 1c).

**Fig 4:**
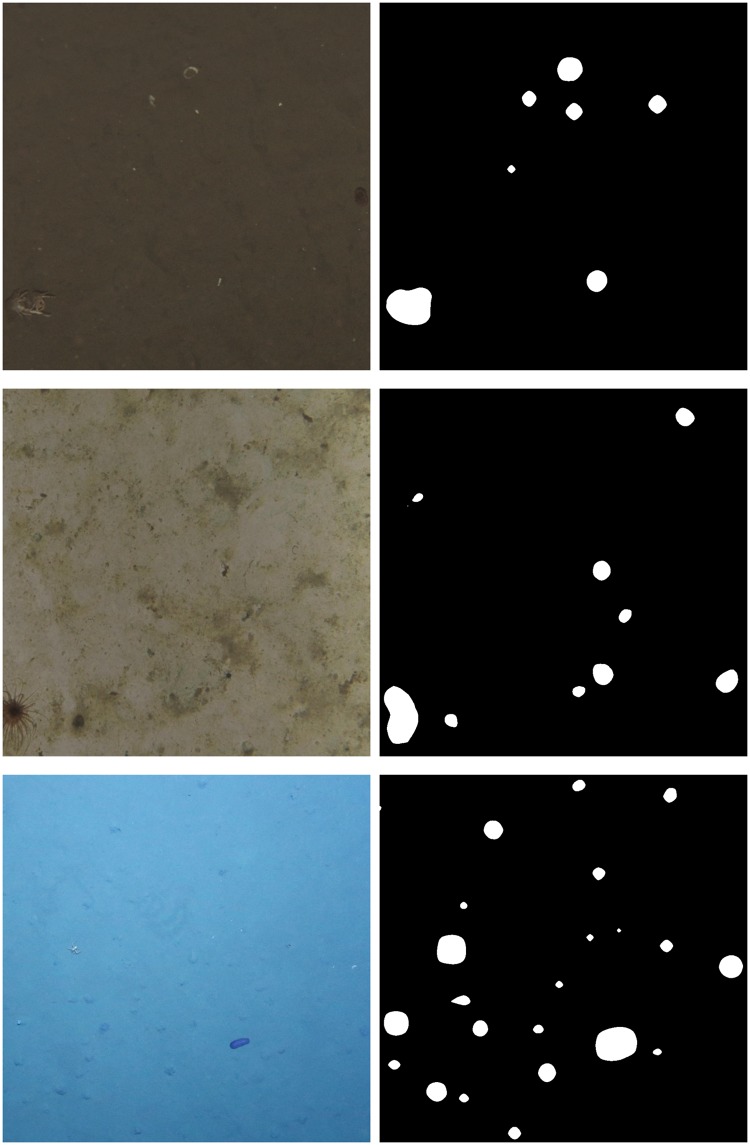
Crops of example images *I*_*i*_ (left column) and their thresholded novelty maps *N*_*i*_ (right column) for the three image datasets JC77 (top row), PAP (middle row) and SO242 (bottom row).

To get a binary segmentation between background and interesting regions, a threshold has to be applied to the novelty map. We empirically determined the threshold *t*_*k*_ to be the mean of the 99-percentiles of the novelty maps of an image cluster *U*_*k*_ (see Eq 5), where P_99_(*N*_*i*_) is the operation that determines the 99-percentile of a novelty map *N*_*i*_. Examples of thresholded novelty maps can be found in Fig 4.

$$\begin{array}{c} t_{k}=\frac{1}{|U_{k}|}\sum_{i:I_{i}\in U_{k}}{\text{P}}_{99}\left(N_{i}\right) \end{array}$$ (5)

The binary segmentation of all novelty maps *N*_*i*_ is used to extract the set of training proposals *T*_*p*_ from all images. Each training proposal *y*_*j*_ ∈ *T*_*p*_ is a part of an image, cropped to the minimum square bounding box of an interesting region (see Fig 1d). We set the minimum edge length of the bounding box to 30 pixels to ensure a certain size of the training proposals, even if the interesting region is smaller. We define the cumulated novelty scores of the pixels of an interesting region as the novelty score *η*(*y*_*j*_) of the corresponding training proposal.

### Stage II: Manual filtering and refinement of training proposals

A considerable amount of the training proposals in *T*_*p*_ may show patterns that do not represent anything of interest for the domain experts. Thus, we apply a quick manual filtering step, where a human observer selects only those training proposals that contain OOI. The filtering has been implemented as single patch classification as defined in the RecoMIA guidelines [3], so only the isolated image regions of the training proposals are displayed to the human observer instead of the complete images. This saves the time the human observer would need to screen the complete images for multiple regions of interest. For each training proposal, the human observer has to determine if it contains (part of) an OOI or not. To accelerate manual filtering, the label review grid overview tool Largo of BIIGLE 2.0 is used (see S1 Fig in the supporting information). Training proposals *y*_*j*_ are displayed in a regular grid in descending order of their novelty scores *η*(*y*_*j*_). This allows human observers to spot OOI very quickly and to review a large number of training proposals in a very short time (see Fig 1e). The sorting by novelty score is a similar technique than the saliency ranking described by [7]. Starting from the training proposals with the highest novelty score, a human observer can stop reviewing once a sufficient amount of training proposals have been selected. In this work we define 600 as the limit for the required number of selected training proposals for each object class.

The performance of an FCN-based instance segmentation method like Mask R-CNN is crucially dependent on the quality of the training dataset. If the samples in the training dataset are of low quality, i.e. with many discrepancies between interesting and non-interesting image regions, the performance of instance segmentation may be very poor. To obtain a set *T*_*s*_ with training samples of appropriate quality from the training proposals *T*_*p*_, a manual refinement step is performed after the filtering. The filtered training proposals are shown to a human observer, each with a suggested centroid and size (i.e. a circle) that marks the OOI. The observer can modify the circle position or size so it closely fits the position and size of the OOI (see Fig 1f). To further accelerate the refinement, we use the volume label review tool Volare of BIIGLE 2.0 (see S2 Fig in the supporting information). With Volare, the viewport of the annotation tool jumps directly from one circle to the next, saving the time a human observer would need to look for and zoom in to each circle on an image.

### Stage III: Instance segmentation with Mask R-CNN

The filtered and refined training proposals are used to build a dataset of training samples *T*_*s*_ for Mask R-CNN (see Fig 1g). This training dataset differs in fundamental aspects from datasets that are typically generated to train FCNs for instance segmentation. In the ideal case, all OOI are marked in all images of the training dataset. In our case, some OOI may have been missed in the novelty detection stage, so these would be falsely labeled as background. To reduce the probability of encountering such falsely labeled OOI during training, we crop a 500 × 500 pixel region around each filtered and refined training proposal *y*_*j*_ and use these crops instead of the whole images as training samples *χ*_*j*_ ∈ *T*_*s*_. Another difference are the circle shapes that are used for fast manual refinement. In a typical training dataset, the annotated segmentation of an OOI is not limited to a particular shape but aims to segment the object as accurately as possible. Here, we define all pixels inside the circle to belong to the OOI, which may include some pixels that actually do not belong to the OOI.

Due to the fixed number of images that are considered to generate a training dataset, there may be only a few hundred or less samples of a particular class of OOI. For comparison, in datasets like MS COCO [6] there are many thousands of instances for each object class. To increase the number of object instances that are available for training, we boost the training dataset (see Fig 1h). Details on the boosting we apply can be found in the supporting information (see S2 Text).

We utilize a freely available TensorFlow implementation of Mask R-CNN [22] in the archived version of [23]. This implementation differs in a few aspects from the original paper [11]. Input images are resized to support training in minibatches, training bounding boxes are generated on the fly and the learning rate is reduced to 10^−3^ (see [23] for details). In MAIA, the training is performed with the set of boosted training samples (see Fig 1i) and the default configuration of the Mask R-CNN implementation. The model is initialized with weights from pre-training with MS COCO [6]. First, all layers except the ResNet 101 backbone [24] are trained for 20 epochs with a learning rate of 10^−3^, a batch size of 2 and 10^3^ batches per epoch. Then all layers including the backbone are trained for another 10 epochs with a reduced learning rate of 10^−4^ but the same batch size and number of batches per epoch. With this configuration, training takes about eleven hours per dataset on a single NVIDIA Titan X.

Inference is performed by padding each image with zeros so that each dimension is divisible by 64. This is done to guarantee smooth scaling in the six levels of the Feature Pyramid Network [25] and still process the image in its original size. Based on the segmentation masks produced by Mask R-CNN, we define any pixel not belonging to the background class as “interesting”. We take the minimum enclosing circle for each region of connected interesting pixels to get the set *A*_*c*_ of annotation candidates (see Fig 1j).

### Stage IV: Annotation candidate review

To eliminate the false positive detections, which mark regions of the images that show no OOI, the annotation candidates *A*_*c*_ are manually reviewed in the last step. This is done analogously to the manual filtering of training proposals in Stage II. Each annotation candidate is shown as an image patch in a regular grid. A human observer then selects all candidates that are true positives, i.e. those that mark an OOI. This yields the final set *A* of annotations.

## 3 Datasets

We evaluated MAIA on three marine image datasets that were collected in different research projects. From each dataset Γ ∈ {JC77, PAP, SO242}, we extracted 500 random images as training subset *T*^Γ^. For each subset, MAIA was performed to train a Mask R-CNN model. The detection performance of the model was evaluated on another 50 random images as validation subset *V*^Γ^ for each dataset. The images of the validation subset have been fully annotated using “traditional” methods.

### JC77 dataset

**Fig 5:**
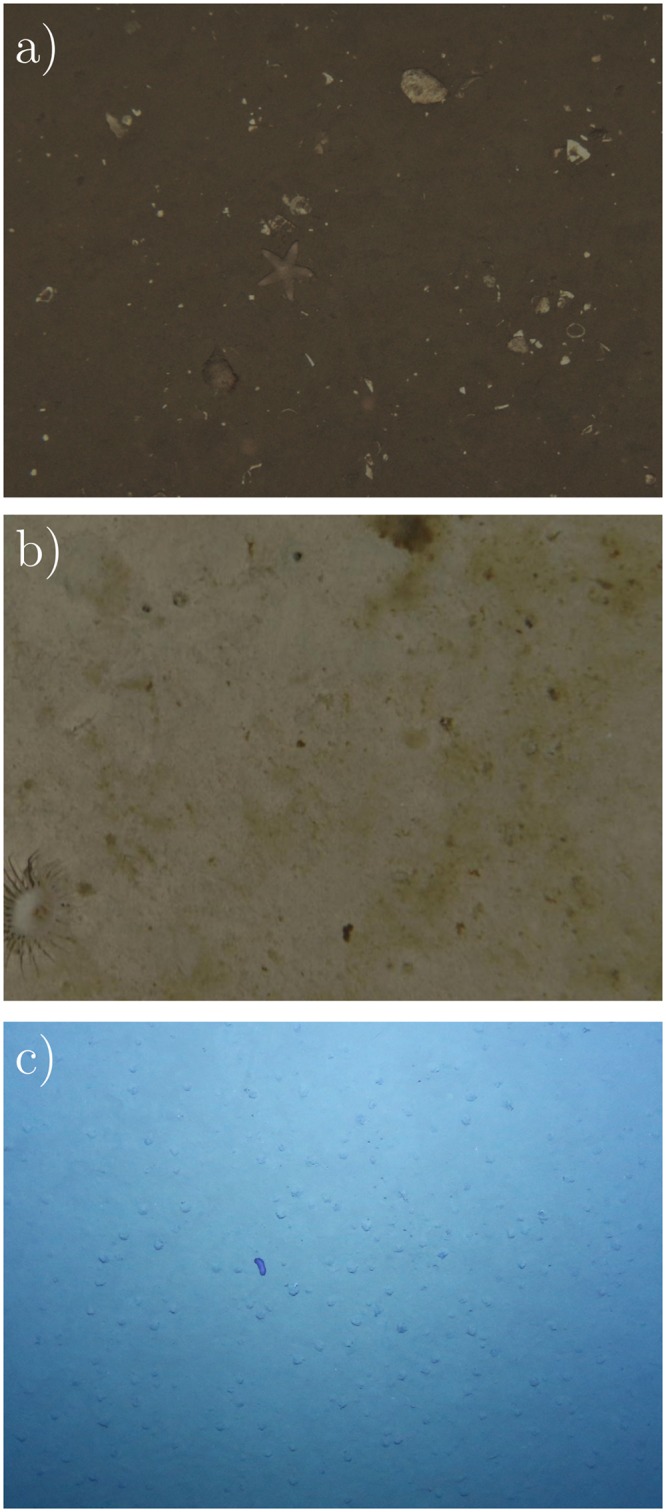
Crops of example images of the JC77 (a), PAP (b) and SO242 (c) datasets. Note the rocks and scattered shell fragments that are no OOI in JC77 (a) and the irregular illumination in SO242 (c).

The images of the first dataset were captured in the Central North Sea near the Sleipner CO2 storage site [26]. It comprises 6321 images of size 2448 × 2048 pixels (see Fig 5a). The images were captured at a depth of 77 m with a target distance to the seabed of 3.0 m. Traditional expert annotation took 166 min for a subset of 125 images, which are 79.68 s ⋅ image^−1^. The two different object classes “shell” (see Fig 2 column 1) and “animal” (see Fig 2 column 2) were annotated with a total of 278 annotations in the 50 images of the validation subset *V*^JC77^.

### PAP dataset

The second dataset contains images from the Porcupine Abyssal Plain (PAP), located southwest of the UK in international waters [27]. The dataset is composed of 3708 annotated images, each of which is a mosaic of ten single images. The images were captured in 4600-4900 m depth with a target distance to the seabed of 3.2 m. 57 different object classes were annotated by experts (for examples see Fig 2). Traditional expert annotation took 480 min for a subset of 243 images, which are 118.52 s ⋅ image^−1^. To eliminate the black background and mosaicing artifacts (e.g. the “sawtooth” boundary between images and background [27]), we extracted tiles of 1000 × 1000 pixels that contain no black background from the mosaics (see Fig 5b). The result is a dataset of 36238 tiles (9.77 tiles ⋅ image^−1^). The original mosaics contain a total of 33358 annotations. Owing to overlaps during tile extraction, the final dataset contains 41033 annotations. The 50 images of the validation subset *V*^PAP^ were chosen so that no overlapping tiles between training and validation subsets exist. The images of *V*^PAP^ contain a total of 57 annotations.

### SO242 dataset

The third dataset consists of 809 images with a size of 4096 × 3072 pixels that were extracted from the SO242/1_83-1_AUV10 survey [28] (see Fig 5c). The images of the survey were captured at depths between 3420 m and 4140 m with a target distance to the seabed of 7.5 m. Traditional expert annotation took 87 min for a subset of 75 images, which are 69.6 s ⋅ image^−1^. The 50 images of the validation subset *V*^SO242^ contain a total of 350 annotations. Only a single “interesting” object class was used to annotate all megafauna in the images of *V*^SO242^.

## 4 Results

Here we present the results of the evaluation of the MAIA stages I-IV. First, we show the detection performance of the unsupervised novelty detection using different AEN parameters. Next, we present the timings of the manual filtering and training proposal refinement steps. Third, we show the detection performance of the trained Mask R-CNN model on the validation subset *V*^Γ^ of each dataset. Finally, we determine the average time it took to annotate an image using MAIA compared to a “traditional” annotation method which employs a sophisticated annotation tool like BIIGLE 2.0 but is purely manual.

### Stage I: Novelty detection with AEN

The AEN, as well as Mask R-CNN, produces a pixel-wise segmentation between background and interesting regions for each image. We evaluated the segmentation with **recall** = *TP*_*θ*_(*TP*_*θ*_ + *FN*_*θ*_)^−1^, (*TP*_*θ*_: number of OOI contained in interesting regions, *FN*_*θ*_: number of OOI not contained in an interesting region) and **precision** = *TP*_*ρ*_(*TP*_*ρ*_ + *FP*_*ρ*_)^−1^, (*TP*_*ρ*_: number of interesting regions containing an OOI, *FP*_*ρ*_: number of interesting regions not containing an OOI). From these values we determined the *F*_2_-score [29] for the detection performance (see Eq 6). We chose the *F*_2_-score to put a higher weight on the recall as this was considered reasonable in the research context at hand.

$$\begin{array}{c} F_{2}=\frac{5\cdot \text{recall}\cdot \text{precision}}{(4\cdot \text{precision})+\text{recall}} \end{array}$$ (6)

**Fig 6:**
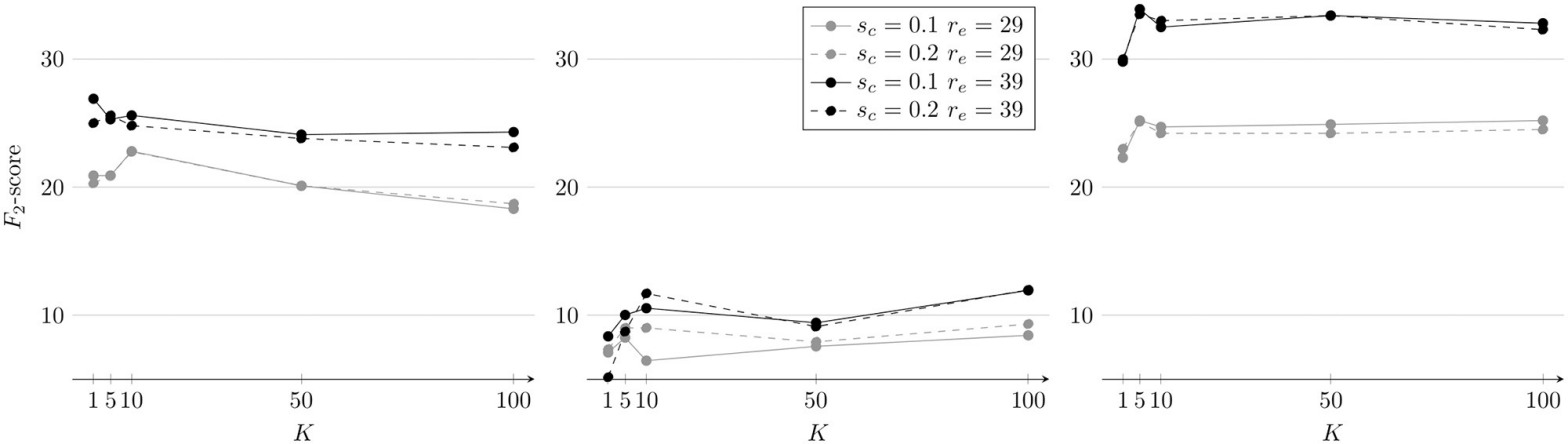
The *F*_2_-scores of each parameter triplet of the AEN parameter search for *V*^JC77^ (left), *V*^PAP^ (center) and *V*^SO242^ (right).

A parameter search was conducted for the number of clusters *K* ∈ {1, 5, 10, 50, 100}, input patch dimension *r*_*e*_ ∈ {29, 39} and latent layer compression factor *s*_*c*_ = *s*/*r* ∈ {0.1, 0.2} of the AEN. For each parameter triplet, we determined the interesting regions of the novelty maps {*N*_*i*_} and calculated the *F*_2_-score for the validation subset *V*^Γ^ of each image dataset. The *F*_2_-scores are visualized in Fig 6 for all three datasets. Detailed values can be found in the supporting information (see S3 Text). On average, the scores of the parameter triplets with *r*_*e*_ = 39 are higher than those with *r*_*e*_ = 29 and a cluster number of *K* = 5 is a good selection for all datasets. The scores of the parameter triplets with a larger *s*_*c*_ do not differ much from those with a smaller *s*_*c*_ and the same *r*_*e*_ but a smaller *s*_*c*_ has a lower computational cost.

### Stage II: Manual filtering and refinement of training proposals

Novelty detection on the 500 images of the JC77 training subset *T*^JC77^ produced a total of 18826 training proposals. These were manually filtered in 61.02 min and refined in 60.17 min which resulted in 440 training samples for the “animal” class and 600 training samples for the “shell” class. Total manual processing of the images of *T*^JC77^ took 14.54 s ⋅ image^−1^ on average.

In the case of the 500 tiles of *T*^PAP^, 2399 training proposals were produced by novelty detection. These were manually filtered in 23.88 min and refined in 22.20 min which resulted in a total of 200 training samples $T_{s}^{\text{PAP}}$. We decided to use the single class “interesting” for the training samples of this dataset, since the number of training samples was too low for a finer taxonomic differentiation. Total manual processing of the images of *T*^PAP^ took 5.53 s ⋅ tile^−1^ which is extrapolated to 54.03 s ⋅ image^−1^ using the ratio of 9.77 tiles ⋅ image^−1^.

For the images of *T*^SO242^, novelty detection produced 30783 training proposals. Manual filtering took only 19.42 min because the limit of 600 training proposals was quickly reached. Refinement of the 600 training proposals took 25.53 min. Total manual processing of the images of *T*^SO242^ took 5.39 s ⋅ image^−1^.

For all datasets, manual filtering and refinement took 24.65 s ⋅ image^−1^ on average, or 3.42 h for the 500 images of a training subset *T*^Γ^.

The training sample datasets have a size of $|T_{s}^{\text{JC77}}|=1040$, $|T_{s}^{\text{PAP}}|=200$ and $|T_{s}^{\text{SO242}}|=600$. However, with the boosting applied, 6 ⋅ 10^4^ unique training samples were used during the 3 ⋅ 10^4^ training steps with batch size 2 for the Mask R-CNN model of each dataset.

### Stage III: Instance segmentation with Mask R-CNN

**Fig 7:**
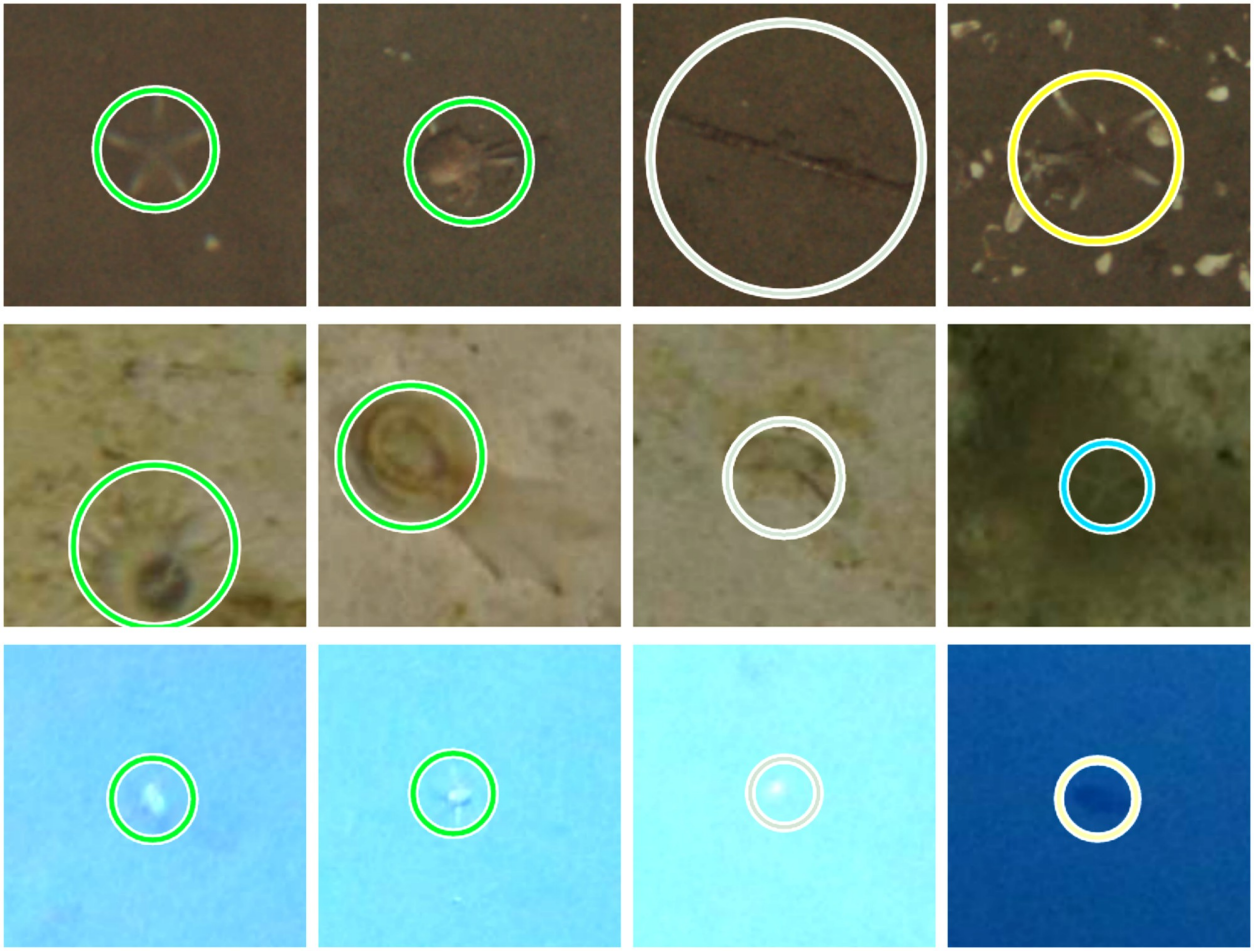
Example annotation candidates (column 1-2 true positives, column 3 false positives) from $A_{c}^{\text{JC77}}$ (top row), $A_{c}^{\text{PAP}}$ (middle row) and $A_{c}^{\text{SO242}}$ (bottom row). Column 4 shows false negatives from the respective validation subset *V*^Γ^.

The binary segmentation between background and interesting pixels produced by Mask R-CNN was evaluated in the same way as the novelty detection in Stage I. For each validation subset *V*^Γ^, precision, recall and *F*_2_-scores were calculated. The results are shown in Table 1. The values for *V*^JC77^ are highest, followed by those for *V*^SO242^. The precision for *V*^SO242^ is almost only half of the precision for *V*^JC77^. The values for *V*^PAP^ are lowest with a precision less than half of the precision for *V*^JC77^. Overall, the instance segmentation achieved an average recall of 84.1% and an average precision of 30.3%. Example annotation candidates for each dataset are shown in Fig 7.

**Table 1:** Detection performance of the trained Mask R-CNN model on each validation subset *V*^Γ^.

Dataset Γ	*F*_2_-score	recall	precision	$|T_{s}^{\Gamma }|$
JC77	78.8	91.7	50.5	1040
PAP	41.1	77.2	14.3	200
SO242	58.0	83.4	26.1	600

### Stage IV: Annotation candidate review

The trained Mask R-CNN model produced 308 to 1094 annotation candidates $A_{c}^{\Gamma }$ for the 50 images of each validation subset *V*^Γ^ (see Table 2). These were reviewed in 2.35 to 16.70 min with an average review time of 18.34 s ⋅ image^−1^. The review time of $A_{c}^{\text{PAP}}$ has been extrapolated from 2.82 s ⋅ tile^−1^ to 27.55 s ⋅ image^−1^. The average review time and the 3.42 h it takes to manually filter and refine training proposals of 500 training images allowed us to build a function *τ*_MAIA_(*n*) for the time it takes to annotate *n* images using MAIA (see Eq 7). Likewise, we were able to build a function *τ*_trad_(*n*) for the time it takes to annotate *n* images using a traditional method (see Eq 8). This function is based on the average annotation time of 89.27 s ⋅ image^−1^ that was measured for the three image datasets.

$$\begin{array}{ccc} \tau_{\text{MAIA}}\left(n\right) & = & 18.34\;\text{s}\cdot {\text{image}}^{-1}\cdot n+12327\;\text{s} \end{array}$$ (7)

$$\begin{array}{ccc} \tau_{\text{trad}}\left(n\right) & = & 89.27\;\text{s}\cdot {\text{image}}^{-1}\cdot n \end{array}$$ (8)

**Table 2:** Number of total annotation candidates $A_{c}^{\Gamma }$, manually selected true positive candidates *A*^Γ^ and the time it took to review them for each dataset. The review time in s ⋅ image^−1^ for $A_{c}^{\text{PAP}}$ has been extrapolated.

Dataset Γ	$|A_{c}^{\Gamma }|$	|*A*^Γ^|	Review time (min)	Review time (s ⋅ image^−1^)	Review time (s ⋅ candidate^−1^)
JC77	501	276	6.20	7.44	0.74
PAP	308	54	2.35	27.55	0.46
SO242	1094	257	16.70	20.04	0.92

**Fig 8:**
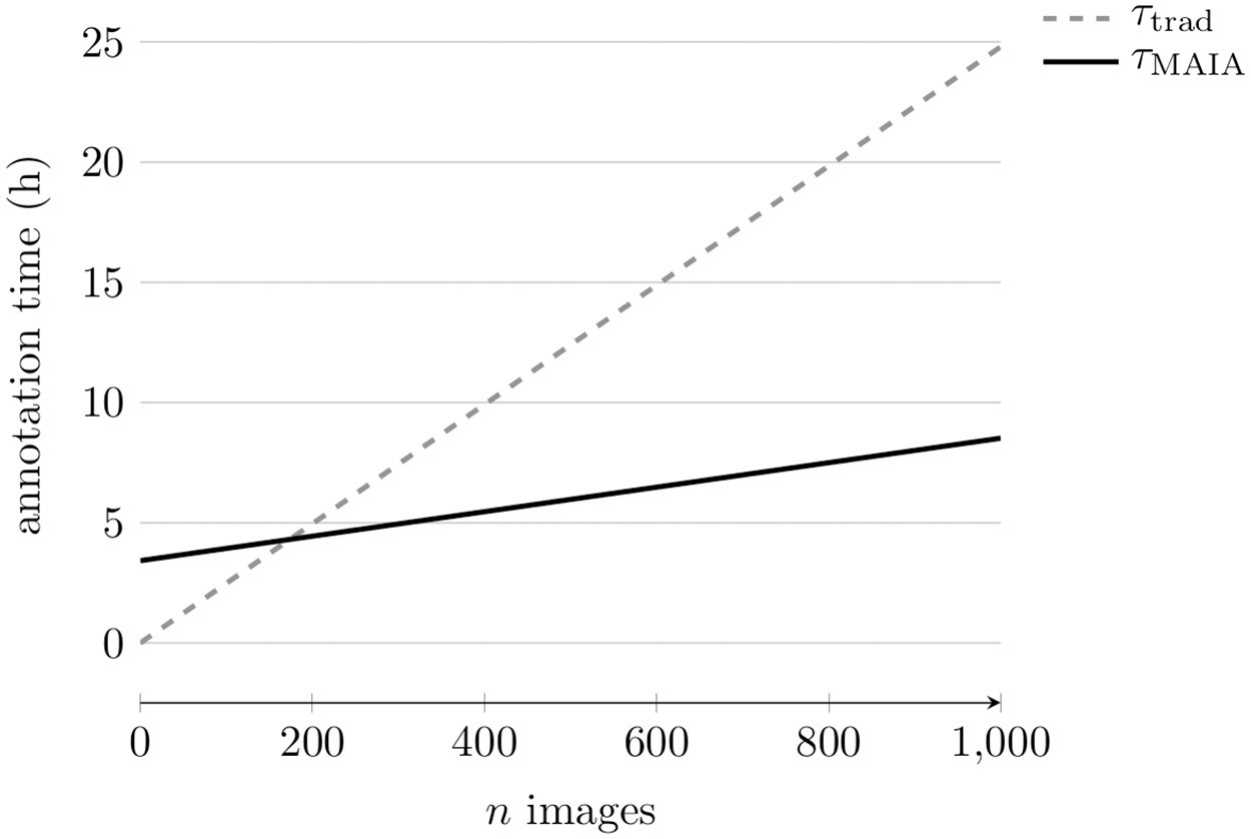
Time it takes to annotate images with a traditional annotation method (*τ*_trad_) compared to the time it takes using MAIA (*τ*_MAIA_).

A plot of the two functions shows that image annotation takes less time with MAIA than with a traditional method when the dataset contains more than 200 images (see Fig 8). The speed-up increases with increasing dataset size.

## 5 Discussion

The results are discussed in the same order as MAIA is described, starting with the AEN-based novelty detection. The results of the AEN parameter search indicate that clustering the images by similar background indeed improves the novelty detection performance. Novelty detection with *K* = 5 clusters yields on average considerably higher *F*_2_-scores than with *K* = 1. However, a higher number of clusters does not further increase the scores. A larger latent layer size, which should enable the AEN to learn a higher number or more accurate image patch properties has no notable effect on the novelty detection performance. A compression factor of *s*_*c*_ = 0.1 seems to be sufficient to learn the visual properties of the seabed which can be briefly described by a dominant color and varying brightness (see Fig 2). The larger input patch size of 39 × 39 pixels resulted in superior *F*_2_-scores compared to the results of the smaller patch size. While it is possible that still larger input patch sizes may further increase the scores, we judged a more exhaustive parameter search to be out of scope of this evaluation. We based this decision on our observations in other scenarios where larger window sizes decrease the performance of machine learning based classification [30]. The novelty detection performance with the presented parameter triplet already resulted in a sufficient number and quality of training proposals to be useful in the annotation method.

Manual filtering and refinement proved to be efficient with the implementation as single patch classification and the available tools of BIIGLE 2.0. Although many thousands of training proposals may sound like an overwhelming number, they can be processed much faster with an average of 24.65 s ⋅ image^−1^ than traditional image annotation with 89.27 s ⋅ image^−1^. Contributing to this speed-up is the ordering of training proposals by novelty scores. Because of the ordering, manual processing of $T_{p}^{\text{SO242}}$ was fastest, although its total number of training proposals was highest among all three training subsets *T*^Γ^.

The size of the set of training samples $T_{s}^{\text{PAP}}$ is small when compared to the number of training samples collected for the other datasets. While a recall of over 77% is still obtained in the final instance segmentation, a higher number of training samples would probably yield better results. This would require more than the 500 tiles of *T*^PAP^ to generate a higher number of training proposals and by this more training samples. Hence, the number of images in a training subset *T*^Γ^ should not be fixed in a future application of MAIA. It should rather be extensible on the fly with new images being added if not enough training samples have been collected.

The Mask R-CNN instance segmentation achieved an average recall of over 84% for the three evaluated datasets. Even with the PAP dataset, where only $|T_{s}^{\text{PAP}}|=200$ training samples were collected, a recall of more than 77% was reached. This is a surprising result for a deep learning model like Mask R-CNN, especially considering the high variability of 57 object classes that are present in the dataset. The fact that a recall of over 77% is still achieved could be a result of the boosting applied to the training samples. The average precision of 30% may seem rather low when compared to other scenarios. In this case, however, a high number of false positive detections has a low cost regarding the review time of the annotation candidates *A*_*c*_. False positives can be dismissed quickly with the Largo tool.

In contrast to image classification or information retrieval in other domains (such as medicine) there is no clear definition of thresholds for a precision or recall to be considered acceptable in marine imaging. However, we can give a heuristic recommendation based on experience. In our experiments, the results were inspected with the Largo tool in a grid overview of 50-100 image patches at the same time. There we considered it helpful if the display of such a set of image patches featured at least five true positives, just to keep and update the visual model(s) for the OOI. Thus, we would argue that 10% is the minimum value acceptable for precision, 10-50% may be considered good and more than 50% may be classified as very good. The recall values should be discussed more conservative as missed OOI can not be compensated as easily as false positive detections. However, it depends on the actual research context of the data collection how to define thresholds there.

The per-instance class labels and bounding boxes that are also produced by Mask R-CNN are ignored which reduces the output to a mere object detection of a single “interesting” class. While this is sufficient for the image annotation method here, one might argue that Mask R-CNN is not the right tool for this task. Hence, we also evaluated a minimized variant of Mask R-CNN that is “cut off” after the region proposal network, omitting the subsequent classification, bounding box refinement and segmentation mask branches. Details can be found in the supporting information (see S4 Text). Although we suspect that the low number of training samples is not sufficient for accurate classification, an evaluation of the class label output of Mask R-CNN remains a topic for future research.

The evaluation of the annotation candidate review points out different characteristics of the image datasets. The images of SO242 have the highest resolution and, with a target distance to the seabed of 7.5 m, the largest footprint of visible objects. Hence, the same number of images can contain a larger number of potential OOI than images with a smaller resolution taken closer to the ground. Combined with the rather low precision of Mask R-CNN for SO242, this contributes to the much higher number of annotation candidates in $A_{c}^{\text{SO242}}$ when compared to the other two datasets. In addition to that, the review time in s ⋅ candidate^−1^ for SO242 is highest among the three datasets. A possible reason for this, again, might be the larger distance to the seabed since smaller objects are harder to recognize.

The review time in s ⋅ candidate^−1^ for $A_{c}^{\text{PAP}}$ is much lower than for $A_{c}^{\text{JC77}}$ although the images of both datasets were taken at a comparable distance to the seabed. This can be explained by differences in object classes between the datasets. Instances of the “shell” class of JC77 (see Fig 2 first column) can sometimes be hardly distinguished from rocks or shell fragments which do not belong to this class. Furthermore, some instances of the “animal” class (see Fig 2 second column) are rather well camouflaged. This is not the case for the majority of OOI in PAP.

The heterogeneity in different properties of the three datasets provides a solid basis to evaluate MAIA. Even the highest review time in s ⋅ image^−1^ stays well below the time that was determined for a traditional annotation method. The annotation time functions *τ*_MAIA_ and *τ*_trad_ show that the constant time it takes to prepare a training dataset for Mask R-CNN is quickly made up for by the much faster process of annotation candidate review when compared to manual scanning of whole images for OOI. For datasets containing 200 or more images, MAIA starts to be faster than a traditional annotation method. In case of the datasets with 550 images that were used in this evaluation MAIA is 2.19 times faster.

An interesting alternative to a visual evaluation of the Mask R-CNN results may be to classify those with another convolutional neural network. Recently, we were able to show that even the problem of strong class imbalance can be solved given the right training and data preparation methods [30].

## 6 Conclusion

We presented MAIA, a novel machine learning assisted method for image annotation in environmental monitoring and exploration. MAIA requires a reduced amount of manual interactions when compared to traditional annotation methods. We have used BIIGLE 2.0 in this work as the interactions required by MAIA can be performed very efficiently with this system. For datasets with more than 200 images, MAIA offers a faster annotation speed with an average recall of 84.1% when compared to traditional methods. The speed-up increases with the size of a dataset and already reaches a factor of 2.19 with datasets of 550 images that were used in our evaluation. Based on these results, we conclude that MAIA is a promising method for image annotation in all environmental monitoring and exploration scenarios with large image collections.

## Supporting information

S1 Text Image clustering. A description of the image clustering that is done prior to the training of the the AEN.
(PDF)

S2 Text Boosting of training samples. The image transformations that are applied during boosting of the Mask R-CNN training samples.
(PDF)

S3 Text AEN parameter search. Detailed results of the AEN parameter search.
(PDF)

S4 Text Mask Region Proposal Network. A description of the architecture, performance evaluation and discussion of the Mask Region Proposal Network (Mask RPN).
(PDF)

S1 Fig The label review grid overview tool Largo of BIIGLE 2.0. Image patches of training proposals or annotation candidates are displayed in a regular grid. Human observers can quickly scroll through all image patches and select those that are of interest with a mouse click.
(TIF)

S2 Fig The volume label review tool Volare of BIIGLE 2.0. The currently focused circle annotation is highlighted at the center of the vieport. Once the human observer finished manipulating the position and size of the circle, the viewport automatically jumps to the next circle.
(TIF)
