# Supplementary material for: MAIA—A machine learning assisted image annotation method for environmental monitoring and exploration
Source: PLoS One. 2018 Nov 16;13(11):e0207498. doi: 10.1371/journal.pone.0207498 (PMC6239313; doi:10.1371/journal.pone.0207498)
Supplement: S1 Text — A description of the image clustering that is done prior to the training of the the AEN. (PDF) [file pone.0207498.s001.pdf]

Image clustering

We perform a clustering of the images prior to sampling the training patches for the autoencoder networks (AEN). First, we downscale each image $I_i = (p_{i,1}, \ldots, p_{i,wh})$ with $w \times h$ pixels to $I'_i = (p'_{i,1}, \ldots, p'_{i,w'^2 wh^{-1}})$ with a width of $w' = 500$ pixels using bilinear interpolation. Then we extract the first two principal components $v'_{i,1}$ and $v'_{i,2}$ for each image I'_i. In addition to that, we compute the image entropy $v'_{i,3}$ for each image, which is based on the Shannon entropy [1] $E(p)$ of a 3×3 neighborhood of a pixel p (see Eq 1).

$$v'_{i,3} = \sum_{a=1}^{w^2 mn^{-1}} E(p'_{i,a}) \quad (1)$$

$$v_{i,j} = \frac{v'_{i,j} - \min_j v'_{i,j}}{\max_j v'_{i,j} - \min_j v'_{i,j}} \quad (2)$$

We scale $v'_{i,j}$ to $v_{i,j} \in [0, 1]$ (see Eq 2) and use $v_i = (v_{i,1}, v_{i,2}, v_{i,3})$ as features for k-means clustering of the images. We choose $K = 5$, based on a parameter search (see Results section). Each image cluster $U_k \subset \{I_1, I_2, \ldots\}$, for $k \in \{1, \ldots, K\}$, is processed independently for novelty detection.

References

1. Shannon CE. A mathematical theory of communication. ACM SIGMOBILE Mobile Computing and Communications Review. 2001;5(1):3–55.
