# Supplementary material for: MAIA—A machine learning assisted image annotation method for environmental monitoring and exploration
Source: PLoS One. 2018 Nov 16;13(11):e0207498. doi: 10.1371/journal.pone.0207498 (PMC6239313; doi:10.1371/journal.pone.0207498)
Supplement: S2 Text — The image transformations that are applied during boosting of the Mask R-CNN training samples. (PDF) [file pone.0207498.s002.pdf]

Boosting of training samples

Boosting is done on the fly during training based on a probabilistic approach. Before each training sample χ_j is considered in a training step, it is transformed to boost(χ_j) as shown in Eq 1, where: $\text{flip}_\text{h}(q, x)$ is horizontal flipping of image x with probability q, $\text{flip}_\text{v}(q, x)$ is vertical flipping of image x with probability q, $\text{rot}(z, y, x)$ is a random rotation between z and y degrees of image x, and $\text{blur}(q, z, y, x)$ is Gaussian blur of image x with a probability of q and a random sigma between z and y.

$$\text{boost}(\chi_j) = \text{blur}(0.5, 0.0, 2.0, \text{rot}(-180, 180, \text{flip}_\text{v}(0.5, \text{flip}_\text{h}(0.5, \chi_j)))) \quad (1)$$
