# Supplementary material for: MAIA—A machine learning assisted image annotation method for environmental monitoring and exploration
Source: PLoS One. 2018 Nov 16;13(11):e0207498. doi: 10.1371/journal.pone.0207498 (PMC6239313; doi:10.1371/journal.pone.0207498)
Supplement: S3 Text — Detailed results of the AEN parameter search. (PDF) [file pone.0207498.s003.pdf]

Autoencoder network parameter search

Fig 1 shows the average F_2-scores of each parameter triplet of the autoencoder network (AEN) parameter search. On average, the the parameter triplet ($K = 5, r_e = 39, s_c = 0.1$) is a good selection for all datasets. Detailed values of the parameter search are shown in Table 1.

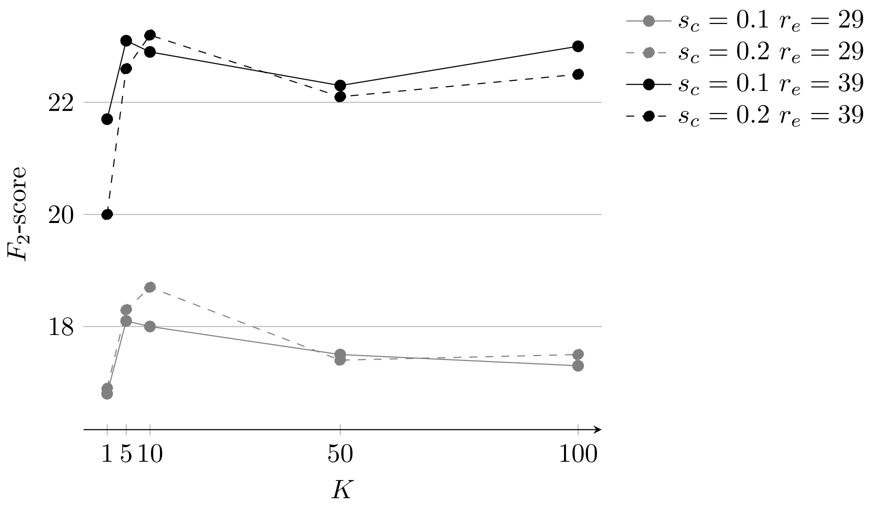

Fig 1. The average F_2-scores of each parameter triplet of the AEN parameter search.

Table 1. F_2-scores of each parameter triplet of the AEN parameter search and each validation subset.

K	s_c	r_e	V^{JC77}	V^{PAP}	V^{SO242}	average
1	0.1	29	20.9	7.08	22.3	16.8
5	0.1	29	20.9	8.24	25.2	18.1
10	0.1	29	22.8	6.44	24.7	18.0
50	0.1	29	20.1	7.56	24.9	17.5
100	0.1	29	18.3	8.42	25.2	17.3
1	0.2	29	20.3	7.34	23.0	16.9
5	0.2	29	20.9	9.00	25.1	18.3
10	0.2	29	22.8	9.00	24.2	18.7
50	0.2	29	20.1	7.91	24.2	17.4
100	0.2	29	18.7	9.30	24.5	17.5
1	0.1	39	26.9	8.34	29.8	21.7
5	0.1	39	25.3	10.01	33.9	23.1
10	0.1	39	25.6	10.54	32.5	22.9
50	0.1	39	24.1	9.40	33.4	22.3
100	0.1	39	24.3	11.93	32.8	23.0
1	0.2	39	25.0	5.15	30.0	20.0
5	0.2	39	25.6	8.71	33.5	22.6
10	0.2	39	24.8	11.69	33.0	23.2
50	0.2	39	23.8	9.10	33.4	22.1
100	0.2	39	23.1	11.97	32.3	22.5
