# Supplementary material for: MAIA—A machine learning assisted image annotation method for environmental monitoring and exploration
Source: PLoS One. 2018 Nov 16;13(11):e0207498. doi: 10.1371/journal.pone.0207498 (PMC6239313; doi:10.1371/journal.pone.0207498)
Supplement: S4 Text — A description of the architecture, performance evaluation and discussion of the Mask Region Proposal Network (Mask RPN). (PDF) [file pone.0207498.s004.pdf]

Mask Region Proposal Network

Our Mask Region Proposal Network (Mask RPN) is based on Mask R-CNN which is "cut off" after the region proposal network, omitting the subsequent classification, bounding box refinement and segmentation mask branches. In addition to that, we define an objectness threshold during inference that discards any region proposal with an objectness score lower than 0.9 prior to non-maximum suppression. We train the Mask RPN model end to end in the same way as the full model described in the Methods section. Training of this reduced model takes less than seven hours per dataset on a single NVIDIA Titan X. We define any pixel that is included in a region proposal bounding box as "interesting" and determine the annotation candidates A_c in the same way as for the full model.

The detection performance of Mask RPN is shown in Table 1. The F_2-scores are lower than those produced by Mask R-CNN for all datasets. Even though the recall is marginally higher for V^{JC77} and V^{SO242}, the precision is much lower. In case of V^{PAP}, the precision stays the same but the recall is greatly decreased.

Table 1. Detection performance of the trained Mask RPN model on each validation subset V^{Γ}.

Dataset Γ	F_2-score	recall	precision	$\lvert T_s^{\Gamma} \rvert$
JC77	73.6	95.3	38.5	1040
PAP	37.5	63.2	14.3	200
SO242	49.9	83.7	19.1	600

The performance comparison of the full Mask R-CNN model and Mask RPN shows that the full model produced superior annotation candidates A_c. Even though the recall is comparable for V^{JC77} and V^{SO242}, the precision is much reduced. In the case of V^{PAP}, the recall is reduced by more than ten percent. The bounding box refinement and classification steps that were removed from Mask RPN seem to play an important role in the ability to learn from very few training samples. However, Mask RPN may still be an option in a context where no great computing power is available, or a quicker runtime is important (e.g. during a cruise on a ship).
