# Supplementary figures and images for: MAIA—A machine learning assisted image annotation method for environmental monitoring and exploration
Source: PLoS One. 2018 Nov 16;13(11):e0207498. doi: 10.1371/journal.pone.0207498 (PMC6239313; doi:10.1371/journal.pone.0207498)

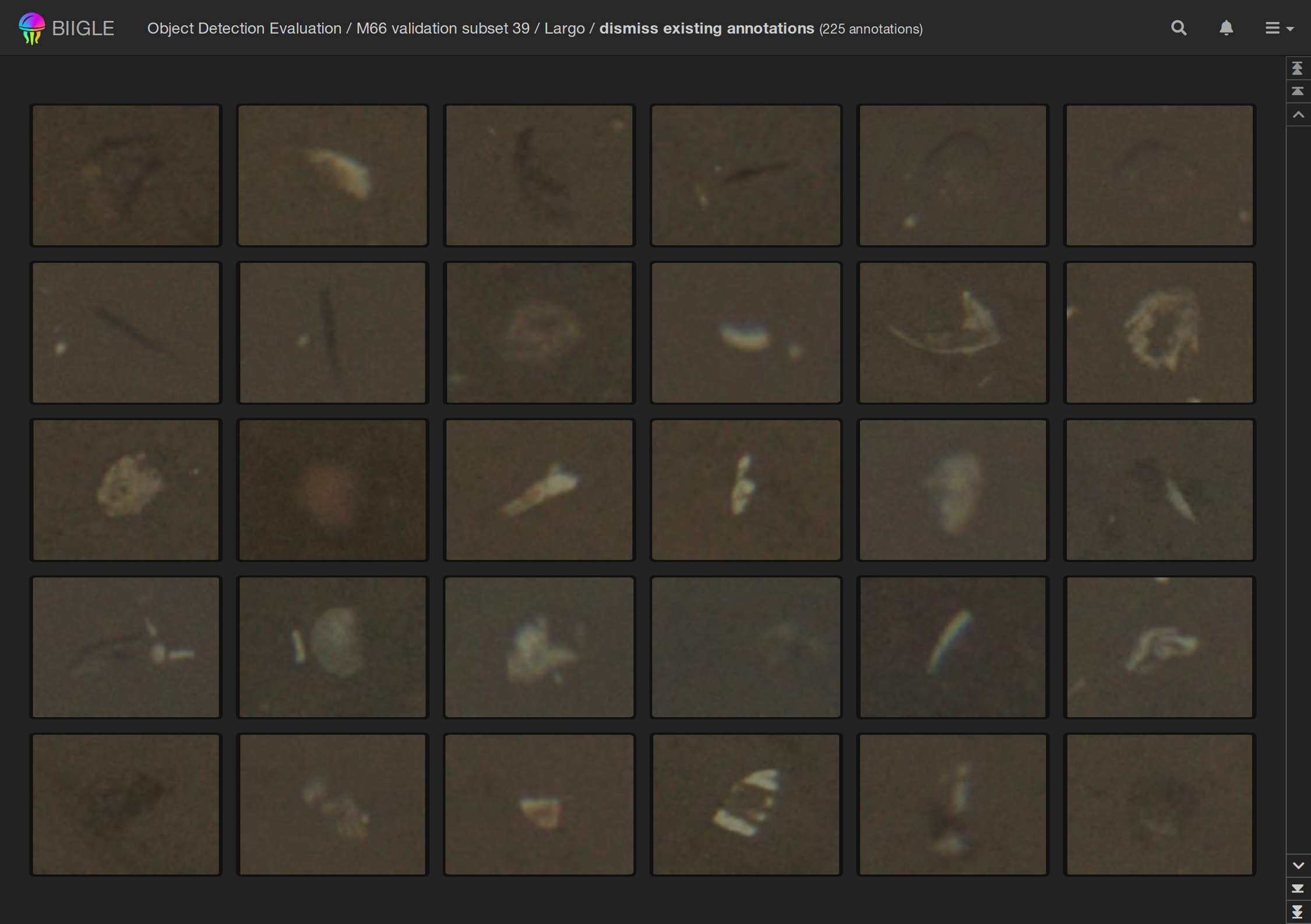

Supplement: S1 Fig — Image patches of training proposals or annotation candidates are displayed in a regular grid. Human observers can quickly scroll through all image patches and select those that are of interest with a mouse click. (TIF) [file pone.0207498.s005.tif]

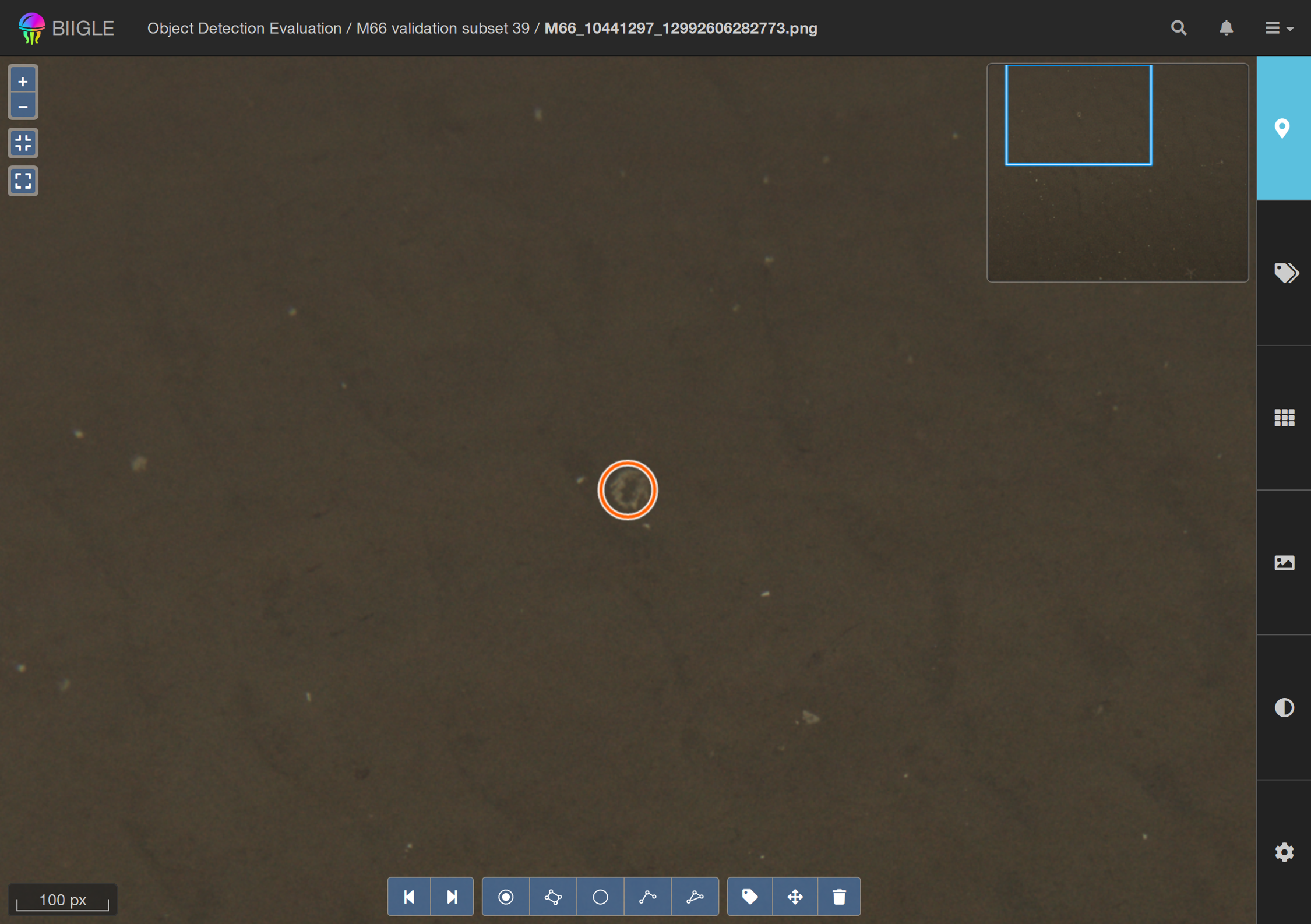

Supplement: S2 Fig — The currently focused circle annotation is highlighted at the center of the vieport. Once the human observer finished manipulating the position and size of the circle, the viewport automatically jumps to the next circle. (TIF) [file pone.0207498.s006.tif]
